# Effects of perioperative hydrogen inhalation on brain edema and prognosis in patients with glioma: a single-center, randomized controlled study

**DOI:** 10.3389/fneur.2024.1413904

**Published:** 2024-07-19

**Authors:** Fan Wu, Tao Liang, Yang Liu, Chenhui Wang, Yongxing Sun, Baoguo Wang

**Affiliations:** ^1^Department of Anesthesiology, Sanbo Brain Hospital, Capital Medical University, Beijing, China; ^2^Department of Anesthesiology, Xuanwu Hospital, Capital Medical University, Beijing, China

**Keywords:** brain edema, glioma surgery, clinical trial, hydrogen inhalation, sleep quality, NRS score

## Abstract

**Introduction:**

Brain edema is a life-threatening complication that occurs after glioma surgery. There are no noninvasive and specific treatment methods for brain edema. Hydrogen is an anti-inflammatory and antioxidant gas that has demonstrated therapeutic and preventative effects on several diseases, particularly in the nervous system. This study aimed to determine the therapeutic effects of hydrogen administration on brain edema following glioma surgery and elucidate its mechanism.

**Methods:**

A single-center, randomized controlled clinical trial of hydrogen inhalation was conducted (China Clinical Trial Registry [ChiCTR-2300074362]). Participants in hydrogen (H) group that inhaled hydrogen experienced quicker alleviation of postoperative brain edema compared with participants in control (C) group that inhaled oxygen.

**Results:**

The volume of brain edema before discharge was significantly lower in the H group (*p* < 0.05). Additionally, the regression rate of brain edema was higher in the H group than in the C group, which was statistically significant (*p* < 0.05). Furthermore, 3 days after surgery, the H group had longer total sleep duration, improved sleep efficiency, shorter sleep latency, and lower numerical rating scale (NRS) scores (*p* < 0.05).

**Discussion:**

In conclusion, hydrogen/oxygen inhalation effectively reduced postoperative brain edema in glioma patients. Further research is necessary to understand the underlying mechanisms of hydrogen’s therapeutic effects. Hydrogen is expected to become a new target for future adjuvant therapy for brain edema.

## 1 Introduction

Brain edema is the pathological swelling of the brain caused by the accumulation of excessive fluid in the brain (1). In patients with tumors, postoperative brain edema is an independent risk factor for worse prognosis and higher mortality (2). Numerous studies have reported a strong correlation between the neuroinflammatory response and the onset and progression of brain edema (3–5). The existing treatments for brain edema lack specificity, mainly requiring invasive procedures and carrying the risk of side effects. These treatments include the use of drugs, invasive surgery, lumbar punctures, etc. (6, 7). Currently, there is a remarkable demand for treatments that are non-invasive, highly efficient, and involve minimal side effects.

Molecular hydrogen has served as both a preventative and therapeutic medical gas for various diseases (8). Due to its proven anti-inflammatory and antioxidant properties, hydrogen has recently become a popular research subject. It has no color or scent, and no side effects of hydrogen have been documented. It has been extensively applied to treat skin cancer, chronic obstructive pulmonary disease (COPD), and sepsis. Yao et al. (9) found that treatment with hydrogen-rich saline improved the inflammatory response and cellular apoptosis in myocardial ischemia/reperfusion (MI/R) via PINK1/Parkin-mediated mitophagy. Zhang et al. (10) discovered that hydrogen could be advantageous in the improvement of mitochondrial function. Yan et al. also revealed that inhaling 2% hydrogen for 3 h may serve as an effective therapeutic strategy for sepsis-induced liver injury in rats. Nishijima et al. (11) further reported that patients with acute brain infarction responded well to hydrogen treatment. These findings suggest that hydrogen gas has broad and universal applications (11). Recent animal experiments investigating hydrogen’s ability to alleviate brain edema in traumatic brain injury (TBI) rats suggest that hydrogen can protect neurons and reduce brain edema in rats (12, 13). However, to our knowledge, no relevant clinical trials have been reported.

A single-center, randomized controlled study was conducted to assess the efficacy and safety of hydrogen therapy on glioma patients. The therapeutic potential of hydrogen in the field of neurology was explored, providing a strong theoretical basis for the future hydrogen applications. This study aimed to reduce hospital stay, accelerate postoperative recovery for glioma patients, and provide a cost-effective solution for managing brain edema.

## 2 Methods

### 2.1 Study design

A randomized controlled clinical trial was conducted at Sanbo Brain Hospital, Capital Medical University (Beijing, China) from August 2023 to October 2023. The first case was recorded on 4 August 2023. The study was approved by the Medical Ethics Committee of Sanbo Brain Hospital, Capital Medical University (Approval No. IRB2020-YX-061-01), and it was registered in the Chinese Clinical Trial Registry (Registration No. ChiCTR2300074362). Written consent was obtained from patients or their family members.

### 2.2 Study participants

The study was conducted on patients undergoing surgery for glioma who met the following inclusion criteria; (1) Class I or II patients classified by the American Society of Anesthesiologists (ASA) guideline; (2) Patients aging older than 18 years; (3) Patients who have undergone surgery for the first time and have not been treated with radiotherapy or chemotherapy; (4) Patients who have signed an informed consent form. The exclusion criteria were as follows: (1) Patients with severe hepatic and renal insufficiency; (2) Pregnant or lactating women; (3) Patients who requested to withdraw during the course of the experiment; (4) Changing treatment plan or refusal of surgical treatment by the patient; (5) An unplanned second surgery or serious complications that might threaten the patient’s life; (6) Participants who were allergic to hydrogen; (7) A history of mental illness; (8) The request of study withdrawal by patients or their family members.

### 2.3 Randomization

Randomization was based on a computer-generated allocation sequence and was performed using a password-protected, encrypted web interface. The 1:1 allocation sequence was stratified by the study site, and permuted, random block sizes of 4, 6, and 8 were utilized.

### 2.4 Procedure

The patients were randomly divided into two groups. One group was the control (C) group, where 33.3% oxygen was administered via a nasal cannula (0.5 L of pure oxygen mixed with 2.5 L of air). The other group, hydrogen (H) group, received 66% hydrogen and 33.3% oxygen via a nasal cannula using a hydrogen inhalation device. A hydrogen/oxygen generator (AMS-H-03; Shanghai Asclepius Meditec Co, Ltd., Shanghai, China) was used. The groups received gas administration for more than 2 h daily. All patients began their respective gas inhalation treatments upon admission.

Patients’ general data, such as age, sex, weight, height, ASA classification, comorbidities, and medication status were collected. Prior to surgery, the tumor’s size, location, and the presence or absence of brain edema were documented. All patients were restricted from consuming clear liquids (e.g., water or electrolyte beverages) for 2 h and solid foods for 8 h before surgery. All patients intravenously received general anesthesia. Invasive arterial monitoring was achieved via successful radial artery puncture. During the intraoperative course, Bispectral Index (BIS) monitoring was maintained at 40–60. The surgical method, operation time, blood loss, urine volume, and fluid volume were also observed and recorded. Postoperative analgesia was provided through intravenous patient-controlled analgesia with sufentanil at 0.03 μg/kg/h and ondansetron at 0.3 mg.

Following surgery, each patient was admitted to the postoperative intensive care unit (ICU), where decisions regarding tracheal extubation were made based on the patient’s condition and the recommendation of the ICU doctor. Upon transfer to the general ward, patients in the H group continued to receive a mixture of 66.7% hydrogen and 33.3% oxygen, and patients in the control (C) group also continued to receive oxygen for the same duration until discharge.

### 2.5 Outcome measures

**Figure 1:**
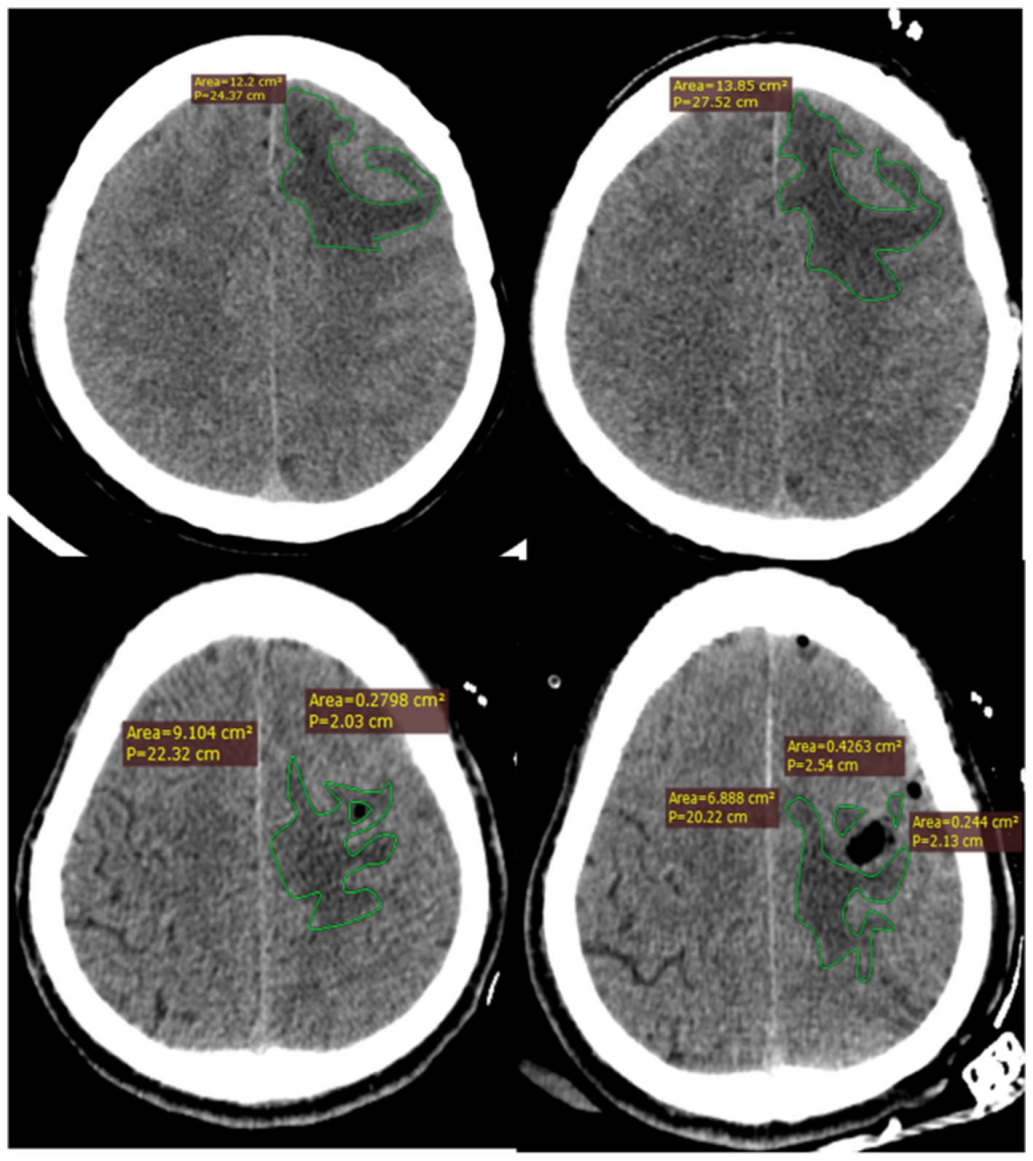
Methods of measuring brain edema using RadiAnt software.

Our primary outcome was to assess the changes in the brain edema volume. The volume of brain edema was evaluated using Radiant DICOM Viewer software. This software enabled us to calculate the edema volume from computed tomography (CT) images obtained at multiple time points: after admission (D1), before surgery (D2), 24 h after surgery (D3), 72 h after surgery (D4), 7 days after surgery (D5), and before discharge (D6). Each patient underwent a CT scan at the specified time points, and the images were uploaded to the Radiant DICOM Viewer. Using the software, we identified and outlined the peritumoral edema on each CT slice. The edema area was manually delineated by two independent, trained neuroradiologists who were blinded to the clinical data and group allocation. The software then calculated the volume of edema by summing the edema areas from each CT slice and multiplying by the slice thickness (0.5 mm). The total edema volume was the sum of the volumes from all slices containing edema. For reliability, both neuroradiologists performed these measurements independently, and the final edema volume for each patient was the average of the two measurements. For patients with irregularly shaped tumors or postoperative cavities, we used a subtraction method, calculating the total volume of the region of interest and then subtracting the tumor or postoperative cavity volume to isolate the edema volume. Any discrepancies between the measurements of the two neuroradiologists were resolved through discussion and consensus. This detailed and systematic approach ensured that our results are both reliable and reproducible (Figure 1).

Secondary outcomes were thereafter monitored. Sleep quality was assessed using SC-500TM system (Beijing Boshi Linkage Technology, Beijing, China). Observations were conducted after admission (D1), before surgery (D2), 24 h after surgery (D3), 72 h after surgery (D4), and 7 days after surgery (D5). The numerical rating scale (NRS) scores were measured 24 h after surgery (D1), 48 h after surgery (D2), and 72 h after surgery (D3). Key serological indices, including C-reactive protein (CRP), white blood cell count (WBC), neutrophil-to-lymphocyte ratio (NLR), and monocyte-to-lymphocyte ratio (MLR), were measured before surgery (D2) and 24 h after surgery (D3). Additional secondary outcomes included length of hospital stay post-surgery, ICU length of stay, ICU intubation time, operation time, postoperative pulmonary complications, postoperative deep vein thrombosis, and other complications.

### 2.6 Evaluation and analysis

Image evaluation and analysis were conducted by two independent neuroradiologists who had received specific training in detecting brain edema on CT scan. Both radiologists were blinded to the clinical data and grouping. The brain edema volume was determined as the average of the volumes calculated by the two radiologists using software. The edema volume was obtained by summing up the edema area in each layer, multiplied by the layer thickness (0.5 mm). Additionally, irregular edema volume was calculated by subtracting the tumor (or postoperative cavity) volume (Figure 1).

### 2.7 Sample size calculation

In our previous studies, the rate of edema regression before discharge was 30%. Additionally, the degree of brain edema regression before discharge with hydrogen inhalation reached 60%. The significance level and the statistical power were set at 0.05 and 80%, respectively. Considering a loss-to-follow-up rate of 0.2, and 100 patients were enrolled (*n* = 50).

### 2.8 Statistical analysis

Categorical variables were presented as the number and percentage of patients. Continuous variables were presented as the mean and standard deviation (SD). A *p*-value of 0.05 was set as the threshold for statistical significance. Numerical variables were analyzed using an unpaired t-test. Categorical variables were analyzed using the χ^2^ test. In order to evaluate the association of mean inhalation duration with brain edema volume and sleep quality, curve estimation method was employed. Statistical analysis was carried out using SPSS 26.0 (IBM Corp., Armonk, NY, United States) and GraphPad Prism 5.0 (GraphPad Software, Inc., San Diego, CA, United States) software.

## 3 Results

**Figure 2:**
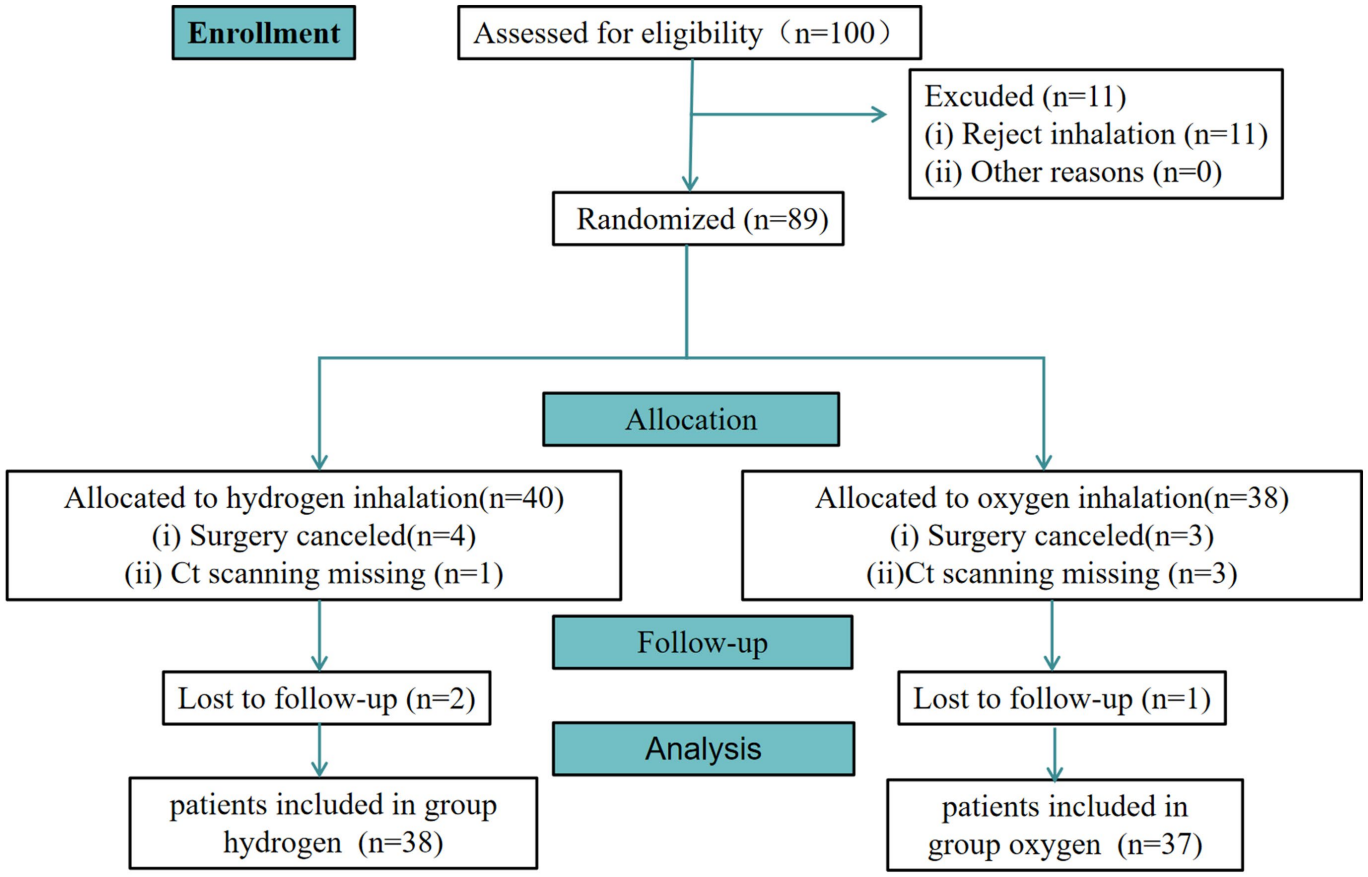
Flow chart of participants inclusion.

From August 2023 to October 2023, a total of 100 adults were assessed for eligibility. Among them, 11 patients rejected to receive inhalation. Besides, 89 patients were randomized to H group (a mixture of 66.7% hydrogen and 33.3% oxygen gases) or C group (a 33% oxygen group). Moreover, 7 patients did not receive surgery. Furthermore, 4 patients missed CT scan. Notably, 75 patients were involved in the primary analysis. Then, 3 patients neglected to complete follow-up sessions. Finally, 75 patients were included in the per-protocol analysis (Figure 2).

Tables 1, 2 show that there were no significant differences between the two groups in terms of preoperative demographic information, intraoperative general state, or tumor location. There were no significant differences in the dosage of mannitol either, before, during, and after the surgery between the two groups (Table 3).

**Table 1:** Baseline characteristics of 75 patients with glioma.

	C group (*n* = 37)	H group (*n* = 38)	*P*
Gender (female, *n*)	16 (43.24%)	20 (52.63%)	0.416
Age (years)	46.18 ± 14.44	45.78 ± 14.59	0.905
Weight (kg)	62.45 ± 10.87	64.47 ± 11.24	0.433
Height (cm)	165.45 ± 7.93	166.92 ± 8.58	0.447
BMI (kg/m^2^)	22.83 ± 3.72	23.15 ± 3.68	0.708
ASA I (*n*)	21 (56.75%)	20 (52.63%)	0.720
Input volume (mL)	4281.25 ± 1086.91	4294.44 ± 1108.05	0.665
Crystal (mL)	3181.08 ± 768.32	3092.10 ± 823.35	0.630
Colloid (mL)	1027.02 ± 310.59	1013.15 ± 318.07	0.849
Blood loss (mL)	400.00 ± 254.40	390.78 ± 255.70	0.876
Urine volume (mL)	2172.97 ± 890.58	2163.15 ± 875.00	0.962
Operation time (h)	5.43 ± 1.62	5.34 ± 1.57	0.808

**Table 2:** Tumor-related information.

	C group (*n* = 37)	H group (*n* = 38)	*P*
Supratentorial tumor	20 (54.05%)	18 (47.36%)	0.642
Subtentorial tumor	17 (45.95%)	20 (52.63%)	0.563
Tumor size (mm^3^)	12.88 ± 8.44	12.77 ± 8.47	0.958

**Table 3:** Perioperative mannitol dosages of 75 patients with glioma.

	C group (*n* = 37)	H group (*n* = 38)	*P*
Preoperative mannitol dosage (mL)	555.40 ± 449.92	609.21 ± 417.78	0.468
Intraoperative mannitol dosage (mL)	221.62 ± 52.09	219.07 ± 53.72	0.788
Postoperative mannitol dosage (mL)	1101.11 ± 422.70	1039.47 ± 444.66	0.153

All patients in the two groups underwent CT scan after admission, before surgery, 1, 3, and 7 days after surgery, and before discharge. Two attending neurosurgeons measured and documented each patient’s brain edema volume, and the average value was thereafter calculated. Before discharge, there was a significant difference (*p* < 0.05) in the volume of brain edema between the two groups. After comparing the edema regression rate on days 3 and 7 after surgery and before discharge between the two groups, it was found that the regression rate in H group was significantly higher than that in C group on the day before discharge (*p* < 0.05). Figure 1 illustrates how the volume of cerebral edema was calculated using the Radiant DICOM Viewer software. Table 4 presents the volume of brain edema in the two groups at corresponding time points before and after surgery. The volume of brain edema before discharge was statistically significant (*p* < 0.05; Table 5).

**Table 4:** The volume of cerebral edema of 75 patients with glioma.

	C group (*n* = 37)	H group (*n* = 38)	*P*
Brain edema volume on D1 (cm^3^)	31.11 ± 16.38	29.32 ± 16.93	0.643
Brain edema volume on D2 (cm^3^)	27.48 ± 12.88	26.41 ± 13.91	0.730
Brain edema volume on D3 (cm^3^)	35.37 ± 27.43	31.57 ± 20.84	0.723
Brain edema volume on D4 (cm^3^)	39.56 ± 26.86	41.15 ± 24.06	0.787
Brain edema volume on D5 (cm^3^)	28.30 ± 20.19	25.90 ± 17.21	0.580
Brain edema volume on D6^*^ (cm^3^)	21.44 ± 19.51	11.5 ± 8.75	0.006

*indicates *p* < 0.05.

**Table 5:** Recovery rate of postoperative brain edema.

	C group (*n* = 37)	H group (*n* = 38)	*P*
The rate of edema regression on the 3rd day after surgery (%)	−0.36 ± 0.41	−0.50 ± 0.56	0.216
The rate of edema regression on the 7th day after surgery (%)	0.03 ± 0.54	−0.04 ± 1.017	0.705
The rate of edema regression before discharge^*^ (%)	0.35 ± 0.31	0.54 ± 0.50	0.041

*indicates *p* < 0.05.

When monitoring sleep patterns between the two groups, there were no statistically significant differences in the sleep data following admission. However, patients in H group experienced significant increases in their deep sleep duration and sleep efficiency on D2, D4, and D5 compared with patients in C group (*p* < 0.05; Table 6).

**Table 6:** Comparison of sleep parameters between the two groups.

Time points		C group (*n* = 37)	H group (*n* = 38)	*P*
D1	Sleep duration	388.21 ± 31.52	401.72 ± 42.85	0.123
	Deep sleep duration	55.92 ± 23.58	62.45 ± 29.48	0.292
	Light sleep duration	247.73 ± 26.45	253.89 ± 44.36	0.467
	Sleep efficiency	84.34 ± 18.46	87.02 ± 18.86	0.513
	R-phase time	81.10 ± 3.50	80.59 ± 6.51	0.673
	AHI	12.63 ± 6.13	12.56 ± 4.82	0.960
	Wakefulness frequency	4.47 ± 2.03	4.47 ± 2.03	0.190
	Sleep latency	32.50 ± 17.40	33.00 ± 21.03	0.911
D2	Sleep duration	389.42 ± 37.62	406.18 ± 44.91	0.084
	Deep sleep duration	57.18 ± 24.22	72.67 ± 29.64	0.015
	Light sleep duration	248.71 ± 8.38	250.67 ± 33.33	0.816
	Sleep efficiency	85.31 ± 18.33	86.56 ± 19.69	0.776
	R-phase time	80.26 ± 5.25	80.24 ± 6.89	0.989
	AHI	13.15 ± 5.69	16.62 ± 9.95	0.067
	Wakefulness frequency^*^	4.97 ± 2.47	4.86 ± 2.45	0.020
	Sleep latency	21.68 ± 12.38	30.54 ± 19.10	0.849
D3	Sleep duration	252.68 ± 46.08	240.37 ± 24.11	0.228
	Deep sleep duration	55.57 ± 19.80	62.37 ± 12.91	0.083
	Light sleep duration	252.68 ± 46.08	240.37 ± 24.11	0.153
	Sleep efficiency	80.31 ± 7.07	77.48 ± 6.71	0.470
	R-phase time	80.31 ± 7.07	77.48 ± 6.71	0.080
	AHI	16.21 ± 3.65	16.91 ± 5.21	0.497
	Sleep latency	34.18 ± 20.37	37.18 ± 19.35	0.142
	Wakefulness frequency	5.10 ± 2.31	6.08 ± 3.31	0.664
D4	Sleep duration^*^	380.37 ± 11.160	416.55 ± 25.10	0.002
	Deep sleep duration	65.33 ± 35.41	86.60 ± 29.23	0.200
	Light sleep duration	232.00 ± 40.88	239.89 ± 28.71	0.649
	Sleep efficiency^*^	70.62 ± 10.55	91.33 ± 24.38	0.042
	R-phase time	76.66 ± 13.30	81.59 ± 21.436	0.326
	AHI	18.33 ± 8.54	17.62 ± 10.12	0.878
	Wakefulness frequency	4.66 ± 2.87	4.37 ± 2.50	0.827
	Sleep latency	41.37 ± 21.23	25.00 ± 19.67	0.120
D5	Sleep duration^*^	359.00 ± 62.06	411.90 ± 31.47	0.029
	Deep sleep duration	69.80 ± 19.34	78.11 ± 32.63	0.503
	Light sleep duration	231.11 ± 40.92	233.90 ± 34.99	0.875
	Sleep efficiency^*^	70.10 ± 8.97	93.11 ± 24.53	0.013
	R-phase time	86.55 ± 30.43	86.90 ± 29.49	0.980
	AHI	15.90 ± 13.05	23.55 ± 11.39	0.193
	Wakefulness frequency	6.30 ± 5.12	4.44 ± 2.50	0.339
	Sleep latency^*^	40.60 ± 28.87	16.89 ± 8.08	0.030

*indicates *p* < 0.05.

The NRS score in H group was lower than that in C group on D4, and the difference was statistically significant (*p* < 0.05; Table 7).

**Table 7:** NRS scores of 75 patients with glioma.

	C group (*n* = 37)	H group (*n* = 38)	*P*
NRS score on D3	4.15 ± 1.12	3.94 ± 1.45	0.482
NRS score on D4^*^	2.89 ± 0.55	2.48 ± 0.65	0.008
NRS score on D5	0.54 ± 0.502	0.55 ± 0.05	0.918

*indicates *p* < 0.05.

There was no significant difference in the laboratory assessment results of CRP, WBC, NLR, and MLR between the two groups (Table 8).

**Table 8:** Laboratory test results of 75 patients with glioma.

	C group (*n* = 37)	H group (*n* = 38)	*P*
Preoperative CRP (mg/L)	2.86 ± 3.04	3.16 ± 2.81	0.665
Postoperative CRP (mg/L)	39.25 ± 58.89	40.85 ± 57.96	0.906
Preoperative WBC (×10^9^/L)	6.32 ± 1.68	6.33 ± 1.60	0.998
Postoperative WBC (×10^9^/L)	14.42 ± 4.56	14.43 ± 4.51	0.989
Preoperative NLR	5.19 ± 4.69	5.22 ± 4.61	0.381
Postoperative NLR	16.17 ± 13.71	15.77 ± 13.75	0.994
Preoperative MLR	0.46 ± 0.92	0.68 ± 1.20	0.997
Postoperative MLR	1.64 ± 3.20	1.64 ± 3.15	0.899

Furthermore, the length of hospital stay following surgery, length of stay in the ICU, intubation time, operation time, postoperative pulmonary complications, postoperative deep vein thrombosis, and other complications did not exhibit any significant differences between the two groups (Table 9).

**Table 9:** Complications and other rehabilitation-related indicators.

	C group (*n* = 37)	H group (*n* = 38)	*P*
Postoperative intubation time (h)	2.86 ± 0.80	2.77 ± 0.88	0.641
Duration of drainage tube retention (d)	4.89 ± 1.89	4.63 ± 2.17	0.583
ICU length of stay (h)	5.21 ± 4.71	4.15 ± 3.55	0.275
Length of stay after surgery (d)	14.54 ± 5.90	14.50 ± 5.78	0.976
Postoperative pulmonary complications (*n*)	8 (21.62%)	6 (15.7%)	0.779
Postoperative deep vein thrombosis (*n*)	5 (13.5%)	6 (15.7%)	0.801
Other complications (*n*)	2 (5.40%)	1 (2.63%)	0.615

**Figure 3:**
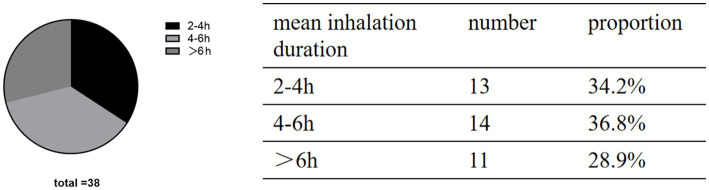
Patients in group H were grouped according to mean inhalation duration.

**Figure 4:**
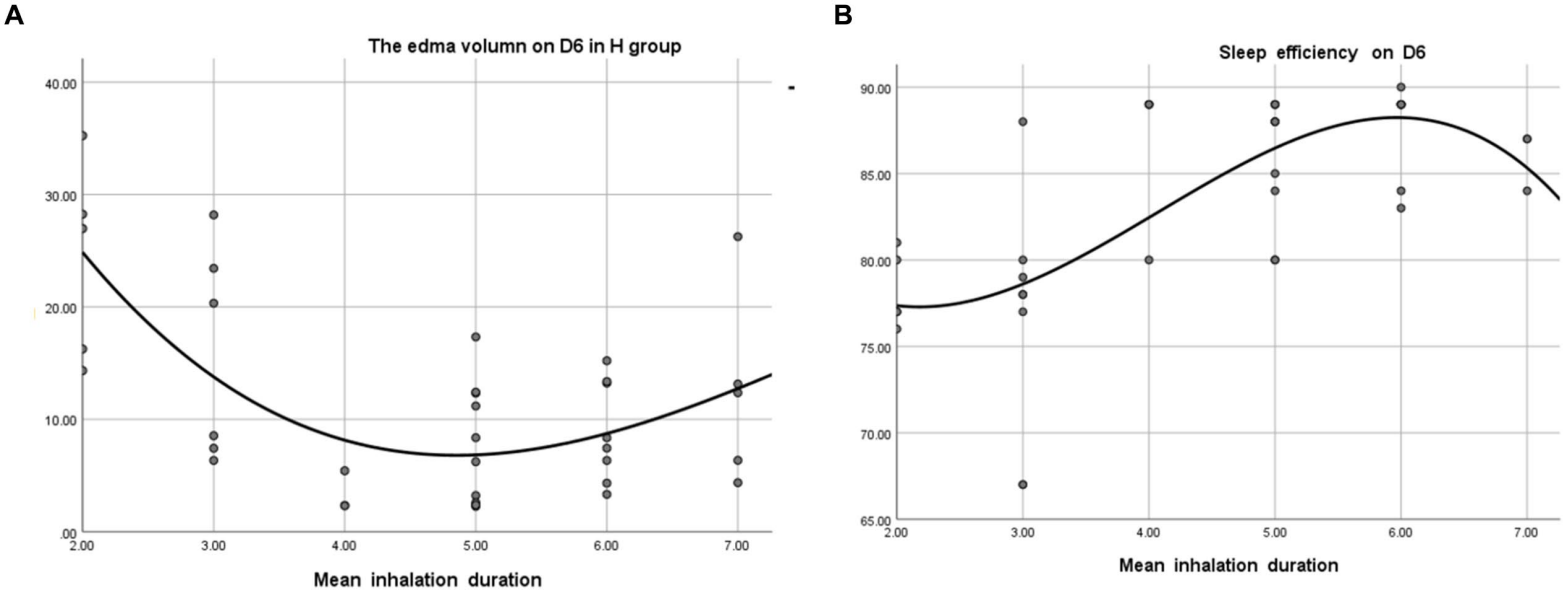
The association of mean inhalation duration with brain edema volume and sleep quality. **(A)** Relationship between the edema volume on D6 and the mean inhalation time in group H.R^2^ = 0.446. **(B)** Relationship between the sleep quality on D6 and the mean inhalation time in group H.R^2^ = 0.487.

Regarding the correlation between inhalation time and sleep and brain edema, correlation analysis was conducted. Patients in the H group were stratified according to inhalation time (Figure 3). It was found that patients who inhaled hydrogen about 5 h/day had the smallest volume of brain edema on the day before discharge. Similarly, patients who inhaled for about 5 h/day also had higher sleep efficiency than those in the other groups (Figure 4).

## 4 Discussion

This study investigated the therapeutic effects of hydrogen inhalation on brain edema following glioma surgery, aiming to address the critical need for non-invasive and effective treatments in this domain. The primary outcome revealed a significant reduction in postoperative brain edema volume in the H group compared with the C group, indicating the potential efficacy of hydrogen therapy in mitigating brain edema.

Although MRI is a commonly used tool for diagnosing brain edema, its early application following brain surgery may accompany by challenges. Fan et al. found that it is reliable to use CT for measuring brain edema volume in the early-stage following surgery (14). Scholars demonstrated that CT can detect and quantify brain edema. They also reported its notable correlation with MRI (15). Therefore, CT scan is convenient, fast, accurate, and reliable, highlighting its broad application.

Growing evidence indicates that hydrogen is a potent therapeutic gas regardless of the concentration being administered. The mixture of hydrogen and oxygen inhalation for the treatment of COVID-19 was recently recommended by the Chinese National Health and Medical Commission (16). Its efficaciousness was also examined in elderly patients with postoperative delirium (POD). The results of the present study revealed that hydrogen inhalation could prevent POD in elderly noncardiac patients by reducing the inflammatory response (17). Furthermore, Xie et al. found that hydrogen treatment could improve the neurological outcome of TBI patients via increasing miR-21 expression (18). Remarkably, the hydrogen-sucking TBI mice had a better neurological prognosis. This may be due to hydrogen’s properties of rapid cellular diffusion, indicating that it can easily diffuse through the blood–brain barrier and relieve brain edema. Its antioxidant properties may play a role in ameliorating brain edema, increasing blood–brain barrier permeability, and reducing brain edema volume (19). Another study proved that hydrogen water could be beneficial in the treatment of early-stage TBI mice (20). Prior research suggested that hydrogen inhalation can significantly inhibit inflammation and reduce the incidence of POD, thereby substantially alleviating postoperative CRP level (21). These data demonstrate that the therapeutic use of hydrogen is extensive and applicable in diverse neurological conditions, including brain edema. Given its broad applicability, low occurrence of side effects, and high permeability, hydrogen could be an extremely effective agent for reducing brain edema volume and enhancing sleep quality.

Even though hydrogen’s anti-inflammatory and antioxidant properties have demonstrated in numerous neurological disorders (22, 23), there are still unanswered questions. In the future research, we will indicate whether hydrogen plays a protective role in neurons by maintaining mitochondrial homeostasis. It has been reported that mitochondrial dysfunction is an important cause of nerve dysfunction after TBI (24), and we have designed *in vivo* and *in vitro* experiments to elucidate the therapeutic function of hydrogen. Furthermore, the present study revealed improvements in sleep quality in the H group, as evidenced by the prolonged deep sleep duration and increased sleep efficiency compared with the C group. While the exact mechanisms underlying these effects warrant further investigation, it is plausible that hydrogen’s modulatory effects on neuroinflammation and oxidative stress contribute to enhanced sleep patterns, reflecting its broader impact on neurological function (11).

However, despite the promising therapeutic effects observed in the present study, it is essential to acknowledge certain limitations. The subjective nature of brain edema volume assessment and the unique design of the hydrogen inhalation machine might introduce potential biases, highlighting the need for rigorous methodology in the future research. Additionally, while hydrogen therapy exhibited efficacy in brain edema reduction and sleep improvement, it did not influence other postoperative outcomes or complications, emphasizing the complexity of glioma surgery recovery.

## 5 Conclusion

The findings of the present study provided a theoretical basis for the effectiveness and safety of hydrogen inhalation therapy in alleviating brain edema. Therefore, hydrogen inhalation could be one possible approach for adjunctive treatment of brain edema.

## Data availability statement

The original contributions presented in the study are included in the article/supplementary material, further inquiries can be directed to the corresponding author.

## Ethics statement

The studies involving humans were approved by the Ethics Committee of Sanbo Brain Hospital, Capital Medical University. The studies were conducted in accordance with the local legislation and institutional requirements. The participants provided their written informed consent to participate in this study.

## Author contributions

FW: Writing – original draft. TL: Investigation, Writing – original draft. YL: Investigation, Writing – review & editing. CW: Writing – review & editing, Software. YS: Writing – review & editing. BW: Writing – review & editing.
